# Correlative analysis between cytotoxic T lymphocyte antigen 4 genetic polymorphisms and head and neck cancer susceptibility: A protocol for systematic review and meta-analysis

**DOI:** 10.1097/MD.0000000000023519

**Published:** 2020-12-11

**Authors:** Bo Lin, Ling Wang

**Affiliations:** a Department of Fundamental Nursing, West Anhui Health Vocational College, Lu’an; b School of Nursing, Wannan Medical College, Wuhu, Anhui Province, China.

**Keywords:** cytotoxic T lymphocyte antigen 4, head and neck cancer, susceptibility, systematic review

## Abstract

**Background:**

Previous published studies have reported the association of cytotoxic T lymphocyte antigen 4 (CTLA-4) genetic polymorphisms with the susceptibility to head and neck cancer, but the results remain controversial. We therefore will conduct a meta-analysis to investigate the relationship between CTLA-4 genetic polymorphisms and head and neck cancer susceptibility.

**Methods:**

We will systematically search case-control studies for potential eligible studies from Cochrane Library, EMBASE, Google Scholar, PubMed, China Biomedical Database, WanFang database, and China National Knowledge Infrastructure (CNKI). Additionally, we will also examine other sources to avoid missing potential trials. Two authors will independently collect and perform the study selection, data extraction, and study methodological quality. Statistical analyses were utilized using STATA 12.0 and RevMan 5.3, and the odds ratios (ORs) with 95% confidence intervals (95% CI) were used to estimate the strength of the association of CTLA-4 genetic polymorphisms with the susceptibility to head and neck cancer.

**Results:**

This protocol study will assess the relationship between CTLA-4 genetic polymorphisms and head and neck cancer susceptibility.

**Conclusion:**

The findings of this study will provide systematic evidence for future guidance developing and clinical decision making in patients with head and neck cancer.

**Ethics and dissemination:**

Ethical approval will not be required as this study is a systematic review.

**Protocol registration number:**

DOI 10.17605/OSF.IO/BFJTZ (https://osf.io/bfjtz/)

## 1 Introduction

Malignant tumors in oral and maxillofacial region, pharynx, and larynx all belong to head and neck cancer. In recent years, due to the multiple effects of heredity and environment, more and more people suffer from HNC, and its mortality rate has ranked the sixth in the world.^[1,2]^ At present, the treatment plan is primarily surgery, supplemented by radiotherapy and chemotherapy. Postoperative communication or dysphagia may occur, seriously influencing the patient's quality of life and low long-term survival. According to current studies, HNC is a multifactorial disease. Excessive smoking and alcohol consumption considered the main risk factors,^[3,4]^ and HPV,^[5]^ chewing betel nut,^[6]^ periodontal disease,^[7]^ and lack of brushing,^[8]^ missing teeth.^[9]^ Up to now, the pathogenesis of HNC has not been specifically studied, and there is more evidence that the susceptibility of HNC is related to gene mutation.^[10]^

Cytotoxic T lymphocyte antigen 4 (CTLA-4, also known as CD152) can affect the activation of T cells and inhibit the anti-tumor response. It is a negative regulatory molecule.^[11]^ The CTLA-4 gene is located on human chromosome 2q33 and consists of 4 exons. Single nucleotide polymorphisms (SNPs) may influence the protein expression and/or the functional activity of CTLA-4.^[12]^ Previous published studies had reported the association of cytotoxic T lymphocyte antigen 4 (CTLA-4) genetic polymorphisms with the susceptibility to head and neck cancer, but the results remain controversial.^[13–16]^ We therefore will conduct a meta-analysis to investigate the relationship between CTLA-4 genetic polymorphisms and head and neck cancer susceptibility.

## 2 Methods

The present protocol report is structured in accordance with the guideline of the Preferred Reporting Items for Systematic Reviews and Meta-Analysis Protocol (PRISMA-P) statement.^[17]^ The present study has been registered on Open Science Framework (OSF, http://osf.io/). The registration DOI number is 10.17605/OSF.IO/BFJTZ.

## 3 Inclusion criteria for study selection

### 3.1 Types of studies

All the included studies were case-control study evaluating the association of CTLA-4 genetic polymorphisms with the susceptibility to head and neck cancer. We will exclude incomplete data or duplicate reports of the same study.

### 3.2 Types of participants

We will include participants with a diagnosis of head and neck cancer. Patients with head and neck cancer will be include regardless of their age, gender, race, smoking status, alcohol status. Control subjects should be defined as healthy volunteers or patients without head and neck cancer.

### 3.3 Types of interventions and comparisons

The intervention group should be defined as CTLA-4 genetic mutation. The control group should be defined as CTLA-4 wild-type.

### 3.4 Types of outcomes

Head and neck cancer risk comparisons, and their 95% confidence interval (95% CI).

## 4 Search methods

### 4.1 Electronic searches

We will systematically search case-control studies for potential eligible studies from Cochrane Library, EMBASE, Google Scholar, PubMed, China Biomedical Database, WanFang database, and China National Knowledge Infrastructure (CNKI) up to October 2020. English- and Chinese-language restriction will be applied.

### 4.2 Search other sources

Additionally, we will also examine other sources to avoid missing potential trials, such as ClinicalTrials.gov (www.ClinicalTrials.gov), reference lists of review articles and all primary studies for additional studies.

### 4.3 Search strategy

We will use the following search terms: (‘Cytotoxic T lymphocyte antigen 4’ OR CTLA-4 OR CD152) AND (polymorphism∗ OR mutation∗ OR variant∗) AND (‘head and neck’ OR oral OR pharyngeal OR oropharyngeal OR nasopharyngeal OR laryngeal OR laryngopharyngeal).

## 5 Data collection and analysis

### 5.1 Selection of studies

**Figure 1:**
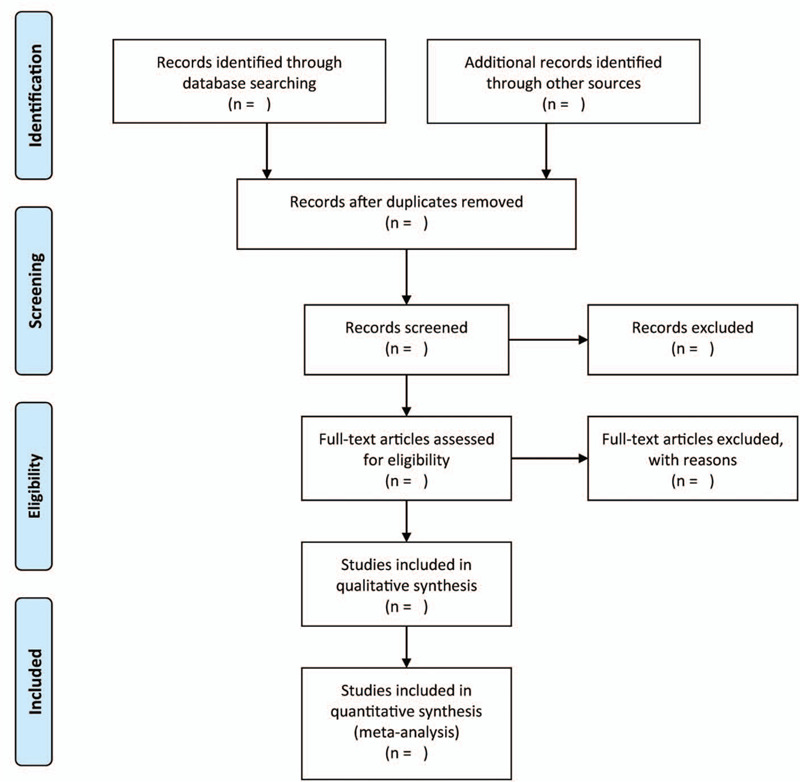
Flow diagram of the literature search.

Two authors will independently screen the titles and abstracts of the records after removal of duplicated studies. Then, the full-text of the selected studies will be obtained for further evaluation. Any disagreement will be settled by consensus. The flow chary of study selection is shown in Figure 1.

### 5.2 Data extraction

Two authors will independently complete research selection and data extraction, and the dispute was settled by discussion. We will extract the following information: the first author's last name, publication year, country and ethnicity of study population, sample size, cancer type, genotyping method, source of controls, genotype distribution of cases and controls, and *P* value of Hardy-Weinberg Equilibrium (HWE) for controls, when *P* < .05 was considered to be inconsistent with HWE.^[18]^

### 5.3 Assessment of study quality

We will use the Newcastle-Ottawa scale (NOS), which is used to evaluate the quality of observational studies, to evaluate the quality of all the included studies.^[19]^ Any disagreement will be settled by consensus. The NOS values arrange from 0 to 9. Studies with a score more than 6 are defined as high quality study.

### 5.4 Measures of treatment effect

We will use the odds ratios (ORs) with its 95% CI to estimate the strength of the association of CTLA-4 genetic polymorphisms with the susceptibility to head and neck cancer.

### 5.5 Assessment of heterogeneity

*Q*-test and *I*^2^ statistics were checked for heterogeneity.^[20,21]^ We first used the fixed effect analysis model to summarize the result. When *P*_*het*_ <.1, *I*^2^ > 50%, we switched the random-effect model.^[22,23]^

### 5.6 Assessment of reporting bias

Publication bias will be estimated by Begg funnel plot and the Egger test (*P* < . 05).

### 5.7 Subgroup and sensitivity analysis

A subgroup analysis of ethnicity and control source was carried out. The HNC metastasis was evaluated using present vs absent. Sensitivity analysis considered the reliability of the results by excluding a study to next study sequentially.

## 6 Discussion

Although previous published studies have reported that the association of CTLA-4 genetic polymorphisms with the susceptibility to head and neck cancer, but the results are still controversial. In addition, no systematic review has been performed to evaluate the association of CTLA-4 genetic polymorphisms with the susceptibility to head and neck cancer. Accordingly, this systematic review will evaluate the association of CTLA-4 genetic polymorphisms with the susceptibility to head and neck cancer. The findings of the present study may provide evidence for clinicians and health-related professionals to make clinical decisions to improve head and neck cancer treatment approach. In the future, large and well-designed case-control studies will be needed to validate our findings.

## Author contributions

**Conceptualization:** bo lin, ling wang.

**Data curation:** bo lin, ling wang.

**Formal analysis:** bo lin, ling wang.

**Funding acquisition:** ling wang.

**Investigation:** ling wang.

**Methodology:** bo lin.

**Resources:** bo lin, ling wang.

**Software:** ling wang.

**Supervision:** ling wang.

**Writing – original draft:** bo lin, ling wang.

**Writing – review & editing:** bo lin, ling wang.
